# Spatial variability of biogeochemistry in shallow coastal benthic communities of Potter Cove (Antarctica) and the impact of a melting glacier

**DOI:** 10.1371/journal.pone.0207917

**Published:** 2018-12-19

**Authors:** Ralf Hoffmann, Francesca Pasotti, Susana Vázquez, Nene Lefaible, Anders Torstensson, Walter MacCormack, Frank Wenzhöfer, Ulrike Braeckman

**Affiliations:** 1 HGF MPG Join Research Group for Deep-Sea Ecology and Technology, Alfred-Wegener-Institut, Helmholtz Zentrum für Polar- und Meeresforschung, Bremerhaven, Bremen, Germany; 2 Marine Biology Research Group, Ghent University, Ghent, Belgium; 3 Cátedra de Biotecnología, Facultad de Farmacia y Bioquímica, Universidad de Buenos Aires; Nanobiotec UBA-Conicet, Buenos Aires, Argentina; 4 Department of Biological and Environmental Sciences, University of Gothenburg, Gothenburg, Sweden; 5 Instituto Antárctico Argentino, San Martín, Provincia de Buenos Aires, Argentina; 6 HGF MPG Join Research Group for Deep-Sea Ecology and Technology, Max Planck Institute for Marine Microbiology, Bremen, Bremen, Germany; Auckland University of Technology, NEW ZEALAND

## Abstract

Measurements of biogeochemical fluxes at the sediment–water interface are essential to investigate organic matter mineralization processes but are rarely performed in shallow coastal areas of the Antarctic. We investigated biogeochemical fluxes across the sediment–water interface in Potter Cove (King George Island/Isla 25 de Mayo) at water depths between 6–9 m. Total fluxes of oxygen and inorganic nutrients were quantified *in situ*. Diffusive oxygen fluxes were also quantified *in situ*, while diffusive inorganic nutrient fluxes were calculated from pore water profiles. Biogenic sediment compounds (concentration of pigments, total organic and inorganic carbon and total nitrogen), and benthic prokaryotic, meio-, and macrofauna density and biomass were determined along with abiotic parameters (sediment granulometry and porosity). The measurements were performed at three locations in Potter Cove, which differ in terms of sedimentary influence due to glacial melt. In this study, we aim to assess secondary effects of glacial melting such as ice scouring and particle release on the benthic community and the biogeochemical cycles they mediate. Furthermore, we discuss small-scale spatial variability of biogeochemical fluxes in shallow water depth and the required food supply to cover the carbon demand of Potter Cove’s shallow benthic communities. We found enhanced mineralization in soft sediments at one location intermediately affected by glacial melt-related effects, while a reduced mineralization was observed at a location influenced by glacial melting. The benthic macrofauna assemblage constituted the major benthic carbon stock (>87% of total benthic biomass) and was responsible for most benthic organic matter mineralization. However, biomass of the dominant Antarctic bivalve *Laternula elliptica*, which contributed 39–69% to the total macrofauna biomass, increased with enhanced glacial melt-related influence. This is contrary to the pattern observed for the remaining macrofauna. Our results further indicated that pelagic primary production is able to fully supply Potter Cove’s benthic carbon demand. Therefore, Potter Cove seems to be an autotrophic ecosystem in the summer season.

## Introduction

Continental shelves comprise only 8% of the global marine realm but are an important component of the marine carbon cycle [1, 2]. Approximately 50% of global benthic mineralization takes place on continental shelves [3]. In shelf areas, benthic mineralization is mainly mediated by the benthic macrofauna community and therefore depends on their biomass, density, structure and functional traits [4, 5], which in turn are influenced by food supply from primary producers and abiotic factors like sediment structure and water temperature [6–8].

The Antarctic continental shelf contributes 1–6% to the entire area of the Southern Ocean [9–11]. However, pelagic primary production over the continental shelf is approximately three times higher than in the open ocean and can reach up to 1600 mg C m^-2^ d^-1^ during the austral summer [9]. The high amount of organic matter input may explain the high benthic faunal biomass found on the Antarctic continental shelf [12]. At shallow, coastal sites at both Signy Island (South Orkney Islands) and Marian Cove (King George Island, Western Antarctic Peninsula), benthic mineralization measured as oxygen fluxes were 12–90 mmol O_2_ m^-2^ d^-1^, and are therefore similar to those of temperate regions [13, 14]. However, apart from these two studies, little is known about the benthic mineralization of organic matter at the sediment–water interface (SWI) in shallow coastal environments of the Antarctic.

The Antarctic summer sea-ice extent and the sea-ice concentration are decreasing at unprecedented rates [15, 16]. Furthermore, glaciers in the West Antarctic and especially on the Western Antarctic Peninsula are melting and retreating [17–19]. These environmental changes can alter physicochemical conditions and benthic communities. A calving-related increase in the ice scour frequency, for example, can cause higher faunal mortality on a local scale [20, 21]. Furthermore, during an ice scour event, the sediment surface is turned over [20, 21] and thereby the seafloor topography is altered [22]. As juveniles or mobile organisms repopulate these areas, ice scour can result in a patchy but diverse benthic community [23], which is continually recolonized, at least locally [24]. In addition, melting glaciers and melting permafrost soils release mainly inorganic particles into marine waters [25], directly or via meltwater streams, and therefore increase the turbidity of the water column [26]. Resuspension events due to ice scour also increase water column turbidity [10, 23]. As a consequence, less light is available for primary producers, which may result in limited primary production, decreased food supply, and ultimately in lower benthic mineralization. Furthermore, particle sedimentation is an important stressor for filter feeders [27] such as common Antarctic ascidians [28] or bivalves [29], which can lead to shifts in the benthic community structure [28, 29]. However, when tidewater glaciers calve and retreat, they open up new ground. Colonization of these newly glacial ice-free areas can increase the local organic carbon supply by primary producers [30] and the local biomass of heterotrophic consumers [31, 32].

At Potter Cove, King George Island/Isla 25 de Mayo, benthic communities have been studied in relation to glacial melt [29–33]. Directly at the glacier front, the biomass of the soft bottom meio- and macrofauna communities was reduced, while at other locations with less influence of glacial melt-related effects, an enriched biomass and a more diverse macrofauna community was found [33]. Biogeochemical fluxes provide an important ecosystem service and are mediated by the benthic community. Therefore, we hypothesize that, due to glacial melt-related effects, biogeochemical fluxes are reduced close to the glacial front compared to less glacial-influenced areas. To investigate this hypothesis, we determined benthic carbon mineralization (represented by diffusive and total oxygen fluxes) and nutrient exchange fluxes across the SWI at the same three locations in Potter Cove where benthic communities where influenced by glacial melt-related effects [33]. Benthic biogeochemical fluxes are, however, influenced by several factors and therefore, we measured key sediment characteristics, parameters representing food supply such as chlorophyll a and organic matter, and the biomass and density of macro-, meio- and prokaryotic assemblages.

Additionally, we addressed the discrepancy between food supply for the benthic community (primary production) and the benthic carbon demand.

## Materials and methods

### Study site

**Fig 1:**
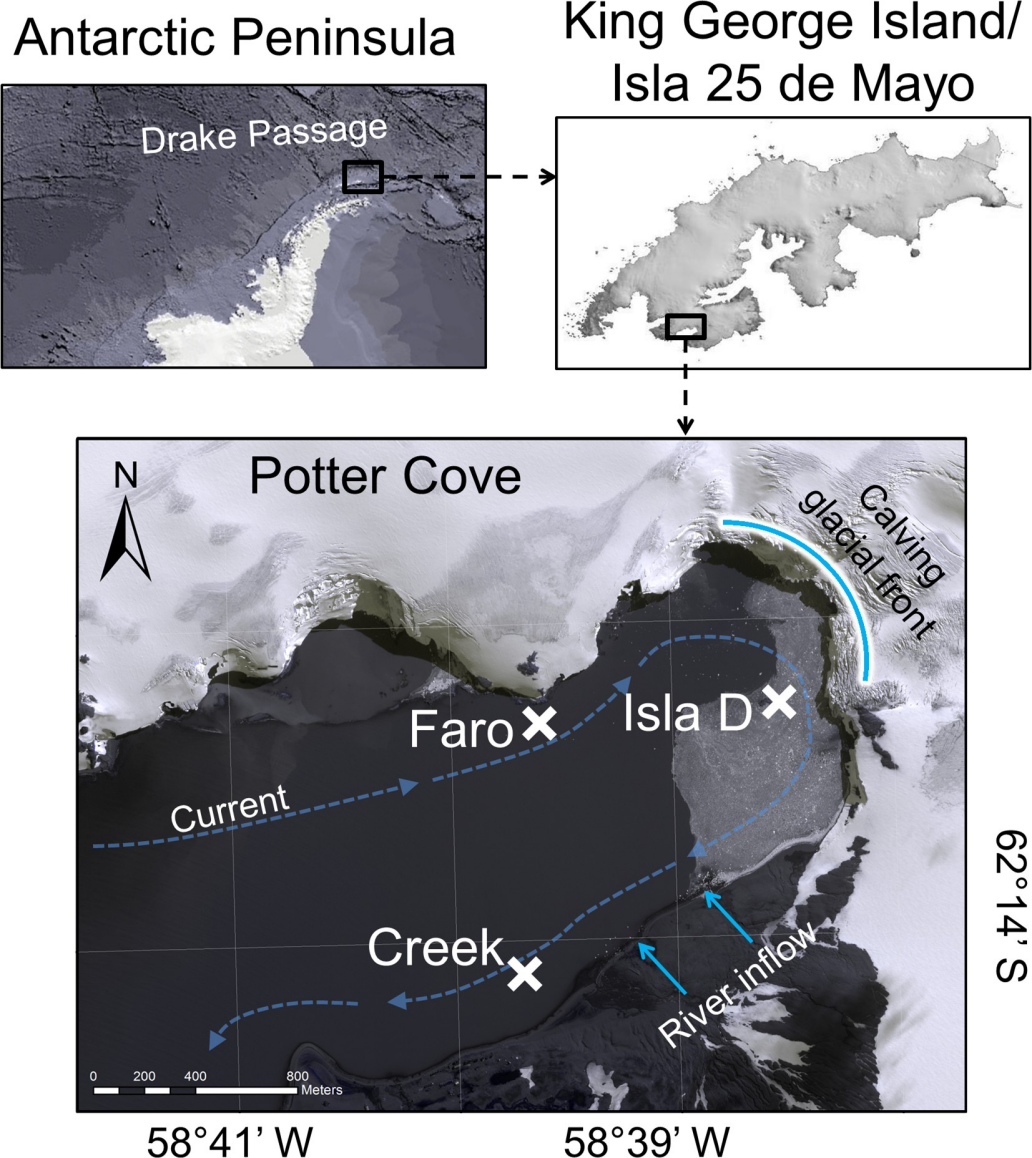
Study site. At Faro, Creek, and Isla D *in situ* measurements and sediment sampling were conducted. The positions of these locations are marked with a cross. The curved, bright blue line marks the front of the Fourcade glacier. The bright blue arrows indicate meltwater streams supplied mainly by waters from glacial, permafrost and snow melting. The dashed blue arrows indicate the direction of the main current in Potter Cove.

Potter Cove is a roughly 3 km long and 1.2 km wide, shallow, fjord-like bay in the south-west of King George Island/Isla 25 de Mayo, an island located at the tip of the Antarctic Peninsula. The cove receives freshwater input from the Fourcade glacier [18] and from seasonal meltwater discharge as a consequence of permafrost and snow melting. The water current flows generally clock-wise around Potter Cove, with an average current speed of 0.03 m s^-1^ [34]. The three locations investigated in the present study (6–9 m water depth, Fig 1, Table 1) are situated in the inner part of the cove and are mainly characterized by soft sediments [33, 35]. The locations, namely Faro, Creek and Isla D, became free of glacial ice between 1988 and 1995, before the 1950s, and before 2005, respectively [18], but are regularly covered by sea ice during winter [36]. The three locations experienced different intensities of glacial melt-related effects. The amount of suspended particulate matter in the water column was highest at Isla D, intermediate at Faro and lowest at Creek [37]. The turbidity at Faro and Creek was similar, while Isla D had a higher turbidity (based on interpolation of data from [30]). The sediment accumulation was lowest at Faro, intermediate at Creek, and highest at Isla D [33]. At 15 m water depth, Isla D, situated directly at the glacier front, is characterized by the lowest macro- and meiofauna biomass, compared to the locations Faro and Creek [33]. The community composition of both macro- and meiofauna differed strongly between the three locations, with the highest trophic diversity found at Faro [33].

**Table 1:** Location, water depth, and date of sampling of the three locations sampled in Potter Cove.

Location	Faro	Creek	Isla D
**Latitude**	62° 13.31' S	62° 14.08' S	62° 13.30' S
**Longitude**	58° 39.36' W	58° 39.43' W	58° 38.30' W
**Depth [m]**	8–9	6–7	8–9
***In situ* measurements and sampling for biogenic compounds [Dates]**	10/02/2015– 12/02/2015	28/02/2015– 01/03/2015	18/02/2015– 19/02/2015
**Pore water sampling dates and number of sediment cores sampled**	09/02/2015: 4 cores	26/02/2015: 2 cores 01/03/2015: 2 cores	18/02/2015: 2 cores 19/02/2015: 2 cores

We measured biogeochemical fluxes at the sediment–water interface and sampled benthic communities and environmental parameters during a field campaign in February and March 2015 (Table 1) at the Argentinean-German Dallmann Laboratory at the Argentinean Carlini research station.

### Sediment properties and biogenic sediment compounds

To measure sediment properties and biogenic sediment compounds, sediment was sampled with 3.6 cm diameter cores in five replicates by SCUBA divers. Sediment subsamples were taken with cut-off syringes (cross-sectional area = 1.65 cm^2^) and sliced in 1 cm intervals down to 5 cm sediment depth. Each interval was analyzed for various parameters including median grain size, porosity, photosynthetic pigments, total carbon, total organic carbon and total nitrogen. Sediment samples for photosynthetic pigments were stored at -80°C. Sediment samples for other parameters were stored at -20°C until analyses were conducted at the home laboratory.

The median grain size was determined with a Malvern Mastersizer 2000G, hydro version 5.40. The Mastersizer used a laser diffraction method and had a measuring range of 0.02–2000 μm. Sediment porosity was estimated after drying sediment samples over a period of at least two days at 105°C. The sediment porosity φ was calculated with the following formula: [38]

$$\varphi =\frac{m_{w}/\rho_{w}}{m_{w}/\rho_{w}+\left(m_{d}-(S\times m_{w})\right)/\rho_{s}}$$

In this equation, *m*_*w*_ is the mass of evaporated water, *ρ*_*w*_ is the density of the evaporated water, *m*_*d*_ is the mass of dried sediment plus salt, *S* is the salinity of the overlying water and *ρ*_*s*_ is the sediment density (2.66 g cm^-3^ [38]). To calculate *m*_*w*_, *ρ*_*w*_, *m*_*d*_ the weight lost by wet sediment samples when dried at 105°C was measured. The uncertainty of the porosity measurement based on the balance precision was <0.01%. Chlorophyll a (*Chl a*), phaeophytin (*Phaeo*) and fucoxanthin (*Fuco*) pigment concentrations were determined by HPLC (Gilson) [39]. The bulk of pigments (*Chl a* plus *Phaeo*) was termed chloroplastic pigment equivalents (CPE) [40]. The ratio of *Chl a* to *Phaeo* served as an indicator for the relative age of the material. The total carbon (TC) and total nitrogen (TN) were measured by combustion using an ELTRA CS2000 with infrared cells. The total organic carbon (TOC) was measured using the same method after acidifying the sample (3 mL of 10 M HCl). Total inorganic carbon (TIC) was calculated by subtracting TOC from TC.

### Density, biomass, and bioturbation potential of the benthic community

To determine prokaryotic density, the same sampling and sub-sampling approach was used as for the sediment properties (see above). Each sediment interval was fixed in a 2% formaldehyde/seawater filtered solution and stored at 4°C. The acridine-orange-direct-count method [41] was used to stain prokaryotes in the sub-samples, which were counted with a microscope (Axioskop 50, Zeiss) under UV-light (CQ-HXP-120, LEj, Germany). For each sample, single cells were counted on two replicate filters and for 30 random grids per filter (dilution factor 3992). Prokaryotic biomass was estimated based on the mean prokaryotic cell volume, measured in the first two centimetres with a “New Portion” grid (Graticules Ltd, Tonbridge, UK) [42], converted into biomass using a conversion factor of 3.0 × 10^−13^ g C pm^-3^ [43] and multiplied by the replicate-specific prokaryote density. Each location-specific mean prokaryotic cell volume represents the mean of 100 counted cells.

For the determination of meiofauna density and biomass and for identification of meiofauna taxa, five sediment samples were collected with small sediment cores (Ø 3.6 cm). Sediment samples of the first five centimeters were stored in buffered 4% formaldehyde/seawater filtered solution at 4°C until extraction at the home laboratory. The samples were sieved on 1 mm and 32 μm mesh, centrifuged three times in a colloidal silica solution (Ludox TM-50) with a density of 1.18 g cm^-3^, and stained with Rose Bengal [44]. Afterward, benthic meiofauna was identified to order level and counted. In order to determine the meiofauna biomass, the total organic carbon content of each taxon was measured with a FLASH 2000 NC Elemental Analyzer (Thermo Fischer Scientific, Waltham, USA). Calcifying organisms were acidified prior to the analysis.

The benthic macrofauna was sampled with a Van Veen grab (530 cm^2^ surface area). At each location, four recovered sediment samples were sieved on 1 mm mesh and stored in seawater buffered 4% formaldehyde. In the laboratory, the taxa were identified to the lowest possible taxonomic level (at least family level), counted, weighed, and the Shannon-Wiener diversity index (H’) was calculated in Primer v6.0. Ash-free dry weight (AFDW) was determined by subtracting the ash weight (after combustion at 500°C) from the dry weight (dried for 48 h at 60°C). AFDW was converted into carbon by assuming that 50% of the AFDW was carbon [45]. Van Veen grab sampling results in a strong underestimation of the density of the Antarctic bivalve *Laternula elliptica* (King & Broderip, 1832). Therefore, two transects of eight grids (45 cm × 45 cm) were randomly placed on the seafloor by SCUBA divers and photos were recorded (Nikon D750, rectilinear Nikon 16–35 mm lens, Nauticam underwater housing, two Inon Z-240 strobes). The photos were used to count siphons of *L*. *elliptica* to determine their density and to measure the siphon width (maximum distance between outer edges of the two siphons of one individual) at the three locations. Assuming a linear relationship between siphon width and AFDW, a conversion factor was used to calculate an estimated biomass of *L*. *elliptica*. The conversion factor was calculated using data from the same *L*. *elliptica* population.

Macrofauna abundance (*Ai*) and biomass (*Bi*) were combined with a mobility score (*Mi*, score between 1–4) and sediment reworking score (*Ri*, score between 1–5) of each taxon (S1 Table) to calculate the community bioturbation potential (*BPc*) with the following formula: [46]

$${BP}_{c}=\sum_{i=1}^{n}\sqrt{B_{i}/A_{i}}\times A_{i}\times M_{i}\times R_{i}$$

in which *i* displays the specific taxon in the sample.

### Biogeochemical fluxes

To quantify the *in situ* benthic organic matter mineralization, three transparent and three black chambers (inner diameter 19 cm, height 33 cm) were carefully pushed into the sediment at each location by SCUBA divers, who took special care not to disturb the sediment surface during the procedure. About 15 cm of sediment and 18 cm of overlying water was enclosed. Cross-shaped stirrers powered by a 12 V lead-acid battery mixed the overlying water. The incubation lasted 20–22 h and included light and dark periods. Owing to dive security regulations, we could not conduct sampling after sunset and thus cannot distinguish between day-time and night-time fluxes of biogeochemical molecules. Therefore, the resulting fluxes represent net fluxes. HOBO Pendant^®^ loggers (Onset, Bourne, USA) were placed both *in situ* and on land to record the amount of radiation (150–1200 nm) during the incubation with a temporal resolution of 5 minutes. The transmission of radiation to the seafloor was calculated based on the readings on land and *in situ*. The enclosed overlying water in the chambers was sampled through valves in the chamber lids at the start and end of the chamber incubation, using gas-tight glass syringes. The water samples were kept at *in situ* temperature and in the dark until further processing, which took place within 1.5 h after the samples were taken.

Subsamples were collected to determine the oxygen concentration, the concentration of dissolved inorganic carbon (DIC) and the concentrations of phosphate, ammonium, nitrite, nitrate, and sulfate. Winkler titration was used to determine the oxygen concentration in the water sample in technical duplicates on site (precision = 0.5% [47]). For DIC analyses technical triplicates were poisoned with HgCl_2_ and stored at 4°C until measurement six months later at the home laboratory. DIC samples were analyzed using an autosampler (Techlab, Spark Basic Marathon, relative standard deviation (RSD) ≤ 0.5%, calibration standardsodium bicarbonate, r^2^ of calibration: ≥0.9997, detection limit: <0.1 mM) with a digital conductivity measuring cell (VWR, digital conductivity meter, Germany) [48, 49]. For nutrient analyses, technical triplicates were filtered through a GF/F filter (Whatman, Maidstone, U.K.) and stored at -20°C until analysis. The samples were analyzed with an autosampler (CFA SAN-plus, Skalar Analytical B.V., Netherlands) for ammonium (RSD ≤ 1.5%, calibration standard: ammonium chloride, r^2^ of calibration: ≥0.9984, detection limit: 2 ppb N), phosphate (RSD ≤ 1.0%, calibration standard: potassium dihydrogen phosphate, r^2^ of calibration: ≥0.9987, detection limit: 2 ppb P), nitrite (RSD ≤ 0.8%, calibration standard: sodium nitrite, r^2^ of calibration: ≥0.9999, detection limit: 2 ppb N), and [nitrate + nitrite] (RSD ≤ 2.5%, calibration standard: sodium nitrate, r^2^ of calibration: ≥0.9984, detection limit: 2 ppb N) concentrations [50]. The nitrate concentration was determined by subtracting the nitrite concentration from the [nitrate + nitrite] concentration.

The total oxygen uptake (TOU) by the benthic community during the incubation was calculated using the formula after Glud [51]:

$$TOU=\frac{\delta O_{2}\times V}{\delta t\times A}$$

in which *δO*_*2*_, *V*, *δt* and *A* represent the difference in oxygen concentration, the volume of the overlying water, the difference in time and the surface area, respectively. The volume of the overlying water was calculated by using the average height between the seafloor and the chamber lid, measured *in situ* by diving at five sites at each chamber. The uncertainty of the TOU measurements based on the precision of the oxygen optode and the height of the overlying water in the core was ca. 5.4%. TOU was converted to carbon equivalents (C-TOU) by applying the Redfield ratio of C:O = 106:138 [52]. The same formula for calculating TOU was used to calculate total DIC and total fluxes of specific nutrients, with *δDIC* and *δNutrient* instead of *δO*_*2*_, respectively. The uncertainties of the DIC, phosphate, ammonium, nitrite, and nitrate flux values were 5.4, 5.9, 6.4, 5.7 and 7.4%, respectively (based on standard deviation (SD) of nutrient analyses and precision of height of overlying water measurement). Several total DIC flux values were omitted, as the difference between t_0_ and t_1_ was lower than the method's detection limit of 0.05 μM.

High-resolution *in situ* oxygen profiles were measured using a microprofiler [53, 54]. The microsensors were driven from the water phase into the sediment with a spatial resolution of 100 μm and a temporal resolution of 30 seconds. On the profiler electronic unit, three custom-made electrochemical oxygen microsensors [55] were mounted and calibrated before deployment as previously described [53, 56]. The microprofiler was programmed so that microsensors penetrated the SWI around noon at the same or the following day after the deployment. Running average smoothed profiles [57] were used to calculate the diffusive oxygen uptake (DOU) over the SWI using Fick's first law after Glud [51]:

$$DOU=-D_{s}\times {\left[\frac{\delta O_{2}}{\delta z}\right]}_{z=0}$$

in which *D*_*s*_ is the molecular diffusion coefficient of oxygen in sediments at *in situ* temperature and salinity. The term [*δO*_*2*_/*δz*]_*z* = 0_ is the oxygen gradient at the SWI calculated by linear regression from the first alteration in the oxygen concentration profile over a maximum depth of 1 mm and therefore only encompassed the diffusive boundary layer. *D*_*s*_ = *D*/θ^2^ [58], with *D* as the molecular diffusion coefficient of oxygen in water [59], and θ^2^ = 1-ln(φ^2^) [60] with the porosity φ within the first centimeter of the sediment. As the identification of the diffusive boundary layer can be difficult, we used the factors *D*_*s*_ in the DOU calculation which include porosity. Therefore, DOU values are slight underestimations. The uncertainty of the porosity values is very small (<0.01%) and thus the uncertainty of the DOU value is similar to the precision of the oxygen microsensor (~1%). Due to hidden dropstones or hard-shelled organisms, a few microsensors broke at a very early stage of the profiling, which resulted in a reduced number of calculated diffusive fluxes per location.

For the calculation of the diffusive flux of sulfate, DIC, and nutrients, sediment was sampled with cores (10 cm diameter) with pre-drilled holes at 1 cm intervals that were sealed with diffusion-tight tape. The pore water was extracted using Rhizons (type: core solution sampler, Rhizosphere Research Products, filter pore diameter of 0.1 mm) connected to 10 mL Luer lock syringes. The Rhizons were horizontally inserted into the sediment and pore water was extracted by creating a permanent vacuum in the syringes. The first drops were used to rinse the syringe and then discarded. The extracted pore water was split for sulfate analyses (sample fixed in 5% ZnAc, stored at 4°C), DIC analyses (sample fixed in HgCl_2_, stored at 4°C) and nutrient analyses (frozen at -20°C). DIC and nutrients were analyzed as described above. Sulfate was analyzed by using non-suppressed ion chromatography with the Methrom 761 Compact IC equipped with a Metrosep A SUPP 5 column (Methrom, Herisau, Switzerland). From the resulting depth profiles (S1 Fig), diffusive fluxes were calculated across a specific sediment depth (S2 Table) using the formula after Schulz [58]:

$$Diffusive\;nutrient\;flux=-\varphi \times D_{s}\times {\left[\frac{\delta C_{Nutrient}}{\delta z}\right]}_{z=0}$$

with the mean porosity φ across the specific sediment depth (S2 Table), *D*_*s*_ of the specific molecule [56], and [δC_Nutrient_/δz]_z = 0_ is the nutrient concentration gradient calculated by linear regression across the specific sediment depth. As the uncertainty of the porosity values is very small (<0.01%), the uncertainty of the diffusive nutrient flux value is similar to the total nutrient flux of the same molecule (see above). Due to the freezing and thawing approach and the volatile character of ammonium, we assess our ammonium concentrations as underestimates, even though filtration and freezing is still the favored treatment if samples cannot be measured directly [61]. However, all samples were treated similarly, and the ammonium fluxes are based on the differences between concentrations. Therefore, we assess the presented ammonium fluxes are accurate.

### Statistical approaches

Fluxes were calculated for each chamber using the slope of concentration over time (incubations; total flux) or depth (vertical profile; diffusive flux). Whenever possible, we tested the significance of the slopes, and only significant regressions over time or sediment depth were used in this study. In case only two data points were available for the slope calculation, we assumed a significant increase or decrease by considering the detection limits of each measurement method.

To test whether the light or dark treatment had an influence on the total fluxes, Student's t-tests were performed on the fluxes of black and transparent chambers. In the case of heteroscedasticity, tested with a Levene's test, a Welch two-sample t-test was carried out. The Gaussian distribution of the data was assumed. Since all t-tests indicated that light had no effect on the total fluxes (S3 Table), data from the different chambers were pooled in all further analyses.

To test whether single parameters differed between locations, a one-way ANOVA (type III SS) and a Tukey post hoc test were performed. A Shapiro-Wilk test was performed to test normality of the data, whereas a Levene's test was used to test homoscedasticity. In cases where the data were not homoscedastic, an adjusted one-way ANOVA and a non-parametric Games-Howell post-hoc test [62] were performed to identify locations showing significant differences. When the data were not normally distributed, absolute values of the data were square root transformed and the Shapiro-Wilk test was repeated. In cases where the transformed data still did not meet the assumptions for parametric tests, a non-parametric Kruskal-Wallis test and a post-hoc Bonferroni test [63] were performed to identify significant differences between the locations.

To visualize relationships between measured parameters among Faro, Creek and Isla D, a principal component analysis (PCA) was performed using the following parameters: median grain size, *Chl a*, *Fuco*, TC, TOC, prokaryotic density, meiofauna biomass, macrofauna biomass, BPc, TOU, total phosphate flux and total nitrate flux. All other parameters were excluded from the PCA as they correlated strongly with one of the mentioned parameters (correlation >0.8, Pearson correlation, S4 Table) used within the PCA. This procedure results in a more resilient PCA result.

To identify the parameters best predicting the measured total oxygen and nutrient fluxes, a preselection of predictor parameters was performed, using “glmnet” [64]. The remaining predictor parameters were used in a linear model, checked for multicollinearity, and stepwise-excluded if they exceeded a vif-value of ten. Afterward, the best predicting parameters were identified using backward selection (omitting the least significant variable, rerunning the model, omitting the next least significant variable) until all partial regression coefficients were significant. The parameter and the biogeochemical flux were log-transformed when the residuals of the final model were not normally distributed or other regression assumptions were not met.

For the statistical analyses, we combined the results obtained from Van Veen grab sampling (macrofauna density (excluding *L*. *elliptica*), macrofauna biomass (excluding *L*. *elliptica*) and the BPc (excluding *L*. *elliptica*)) and the *L*. *elliptica* underwater photo survey (*L*. *elliptica* density, *L*. *elliptica* biomass, *L*. *elliptica* BPc)) into one dataset. Due to the different numbers of replicates (four with Van Veen grab sampling, up to 16 with underwater photography), we calculated the location-specific mean *L*. *elliptica* density, biomass and BPc and added these values to each location-specific replicate value of the related parameter. This step altered the total variance of the parameters that we analyzed in this study, but since the overall SD of the *L*. *elliptica* measurements was below that of the univariate macrofauna biomass values, we are confident that the overall statistical results are accurate.

For the PCA and the identification of the TOU and nutrient flux predicting parameter, a dataset without missing values was required. Therefore, only the first four replicates of parameters describing sediment properties, biogenic compounds, benthic community and biogeochemical fluxes were used. Missing values that occurred in the first four replicate values were filled with data from the fifth or sixth replicate. Furthermore, biogeochemical fluxes were expressed as absolute values. Diffusive flux values were excluded from this dataset, as they are a sub-flux of the total fluxes and contributed in the maximum case 13% (diffusive nitrate flux at Isla D) to the total flux.

All statistical tests were performed using R Statistical Software (version 3.4.0, R Core Team, 2017) and the packages “vegan” [65], “CAR” [66], “Userfriendlyscience” [67], “PMCMR” [68], and “glmnet” [64]. Where replicates were available, results are expressed as mean value ± standard deviation.

### Ethics statement

Research conducted here was approved and permitted by the Environmental and Tourism Antarctic Management Program of the National Direction of the Antarctic (Dirección Nacional del Antártico) in the Argentine Republic prior to the field campaign. Sampling was conducted in the Specially Protected Area N° 132 “Peninsula Potter” (under art. 7, Annex V of the Madrid Protocol, Law 25260) according to all regulations in force. No protected species were sampled.

## Results

### Comparison of abiotic and biogenic parameters

During the incubations, the seafloor at the locations Faro, Creek, and Isla D experienced 13.5, 14.5, and 13.5 h of light incidence, respectively. The light transmission to the seafloor (= light incidence at the seafloor divided by light incidence on land) during daytime was 0.5 ± 0.3, 4.4 ± 1.8, and 0.7 ± 0.5% at Faro, Creek and Isla D, respectively.

**Fig 2:**
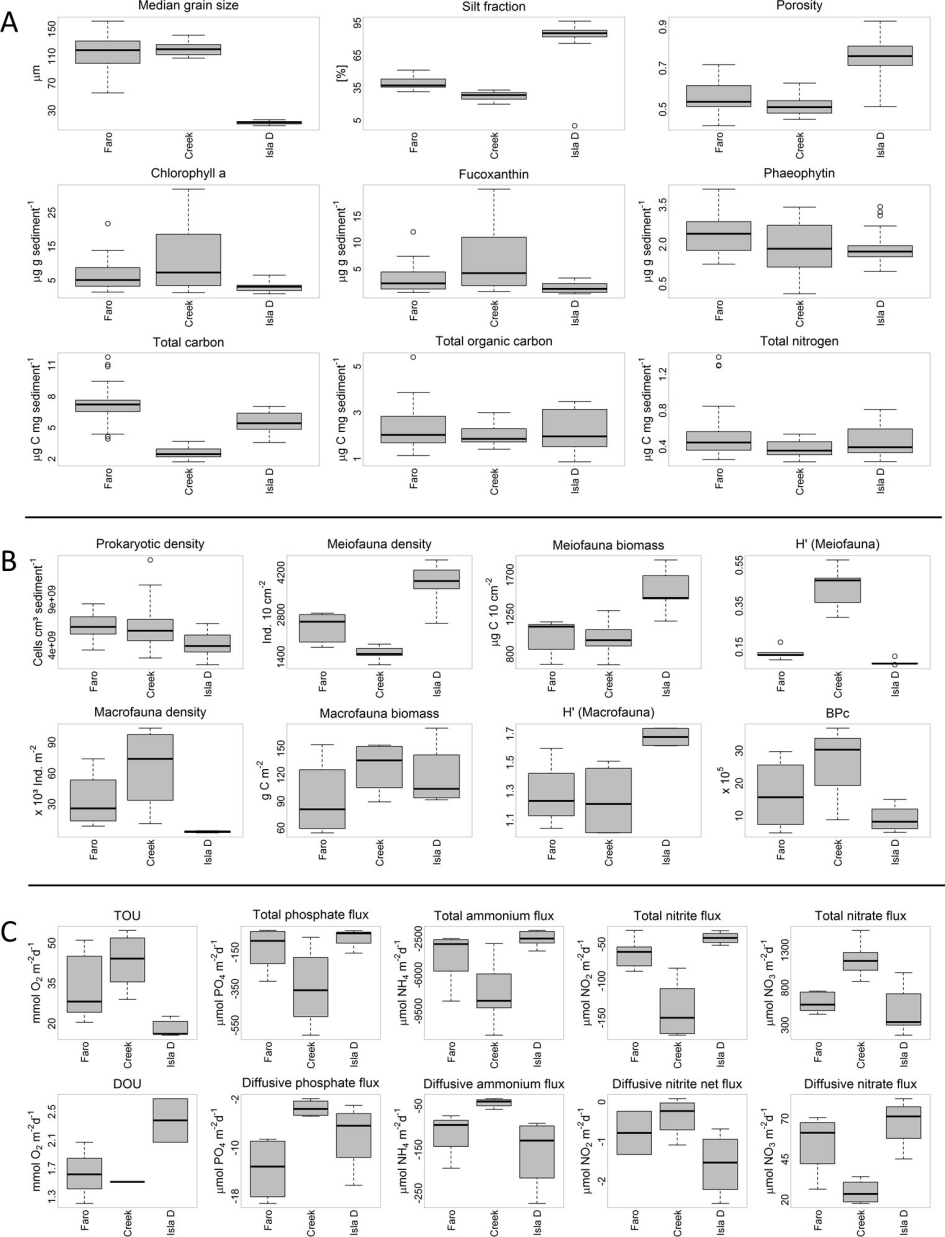
Boxplots of a subset of the measured parameters. Panel A refers to sediment properties and biogenic sediment compounds, panel B refers to fauna community parameters and diversity indices, and panel C refers to total fluxes and diffusive fluxes. H’ of macrofauna was calculated without the results of the *Laternula elliptica* survey.

The median grain size and porosity at Faro were 116 ± 27 μm and 0.56 ± 0.08 over the first 5 cm sediment depth (Fig 2A), respectively, with a silt fraction of 39 ± 5% (S5 Table). Creek had a similar median grain size and porosity (120 ± 9 μm and 0.51 ± 0.05, respectively, Fig 2A) but a lower silt fraction of 28 ± 4% (S5 Table). The sediment at Isla D was finer (median grain size: 20 ± 30 μm, silt fraction 83 ± 12%) and had a higher porosity (0.76 ± 0.12) over the first 5 cm sediment depth (Fig 2A).

The *Chl a* and *Fuco* concentrations at Faro were 6.3 ± 4.6 μg g sediment^-1^ and 3.1 ± 2.6 μg g sediment^-1^, respectively (Fig 2A, S5 Table). The relative age of the biodegradable organic matter, represented by the chlorophyll a to phaeophytin ratio (*Chl a*/*Phaeo*), was 2.3 ± 1.2. At Creek, the *Chl a* concentration was similar (11.3 ± 9.3 μg g sediment^-1^) to Faro, while the *Fuco* concentration (6.6 ± 5.7 μg g sediment^-1^) and the *Chl a*/*Phaeo* ratio (6.9 ± 5.3) were higher (Fig 2A and S5 Table). The *Chl a* concentration at Isla D (3.0 ± 1.4 μg g sediment^-1^) was lower compared to Faro and Creek, whereas the *Fuco* concentration, and *Chl a*/*Phaeo* ratio (1.3 ± 0.9 μg g sediment^-1^, 1.6 ± 0.6, respectively) were similar to Faro but lower compared to Creek (S5 Table). The *Phaeo* concentrations were similar among the three locations Faro, Creek and Isla D (2.4 ± 0.8 μg g sediment^-1^, 1.8 ± 1.0 μg g sediment^-1^, 1.9 ± 0.7 μg g sediment^-1^, respectively).

TC, TIC, TOC, and TN contents over the first 5 cm sediment depth at Faro were 7.2 ± 1.4 μg C mg sediment^-1^, 4.8 ± 0.8 μg C mg sediment^-1^, 2.3 ± 0.9 μg C mg sediment^-1^and 0.5 ± 0.2 μg N mg sediment^-1^ (Fig 2A and S5 Table), respectively, and the organic carbon portion (TOC/TC) was 32 ± 8% (S5 Table). TC, TIC, and TN contents were significantly lower at Creek (2.6 ± 0.5 μg C mg sediment^-1^, 0.6 ± 0.3 μg C mg sediment^-1^and 0.4 ± 0.1 μg N mg sediment^-1^, respectively) compared to Faro, while TOC content (2.0 ± 0.4 μg C mg sediment^-1^) was similar (Fig 2A and S5 Table). Therefore, the TOC/TC ratio was twice as high at Creek (78 ± 11%) compared to Faro (S5 Table). TC (5.5 ± 0.9 μg C mg sediment^-1^), TIC (3.3 ± 0.4 μg C mg sediment^-1^), TN content (0.5 ± 0.2 μg N mg sediment^-1^) and the TOC/TC ratio (39 ± 10%) at Isla D had intermediate values between Faro and Creek, while the TOC content (2.2 ± 0.8 μg C mg sediment^-1^) was in a similar range (Fig 2A and S5 Table).

Median grain size increased with sediment depth at Faro, while no vertical change was observed at the other locations. Porosity, TC, TOC, TN, *Chl a*, *Fuco*, and *Phaeo* concentrations decreased with sediment depth at all three locations, while TIC concentrations did not change over sediment depth. The sulfate concentration in the pore water also did not change over sediment depth and was ~27 mmol SO_4_^2-^ L^-1^ at the three locations (S1 Fig).

### Comparison of benthic community parameters

At Faro, the prokaryotic density was 6.1 ± 1.2 × 10^9^ cells cm^-^^3^ sediment^-1^ and the prokaryotic biomass was 0.26 ± 0.02 mg C cm^-3^ sediment^-1^ (S5 Table). The meiofauna density and the meiofauna biomass were 2368 ± 471 ind. 10 cm^-2^ and 990 ± 190 μg C 10 cm^-2^, while the macrofauna density and macrofauna biomass (excluding *L*. *elliptica*) were 33574 ± 24902 ind. m^-2^ and 56 ± 39 g C m^-2^, respectively (S5 Table). The photo survey revealed an estimated *L*. *elliptica* density of 93 ± 26 ind. m^-2^, an *L*. *elliptica* biomass of 36 ± 9 g C m^-2^ and an *L*. *elliptica* individual biomass of 0.39 ± 0.16 g C ind.^-1^ (S5 Table).

At Creek, the prokaryotic density, meiofauna density, meiofauna biomass, macrofauna density (excluding *L*. *elliptica*), and macrofauna biomass (the latter excluding *L*. *elliptica*; 6.0 ± 2.1 × 10^9^ cells cm^-^^3^ sediment^-1^, 1524 ± 231 ind. 10 cm^-2^, 980 ± 204 μg C 10 cm^-2^, 65612 ± 35948 ind. m^-2^ and 75 ± 26 g C m^-2^, respectively) were similar compared to those reported at Faro (Fig 2B, S5 Table). However, values of prokaryotic biomass, estimated *L*. *elliptica* density, and *L*. *elliptica* biomass (0.22 ± 0.02 mg C cm^-3^ sediment^-1^, 157 ± 44 ind. m^-2^, 54 ± 16 g C m^-2^, respectively) were significantly higher at Creek compared to those at Faro, whereas the *L*. *elliptica* individual biomass (0.34 ± 0.14 g C ind.^-1^) was significantly lower (S5 Table).

**Fig 3:**
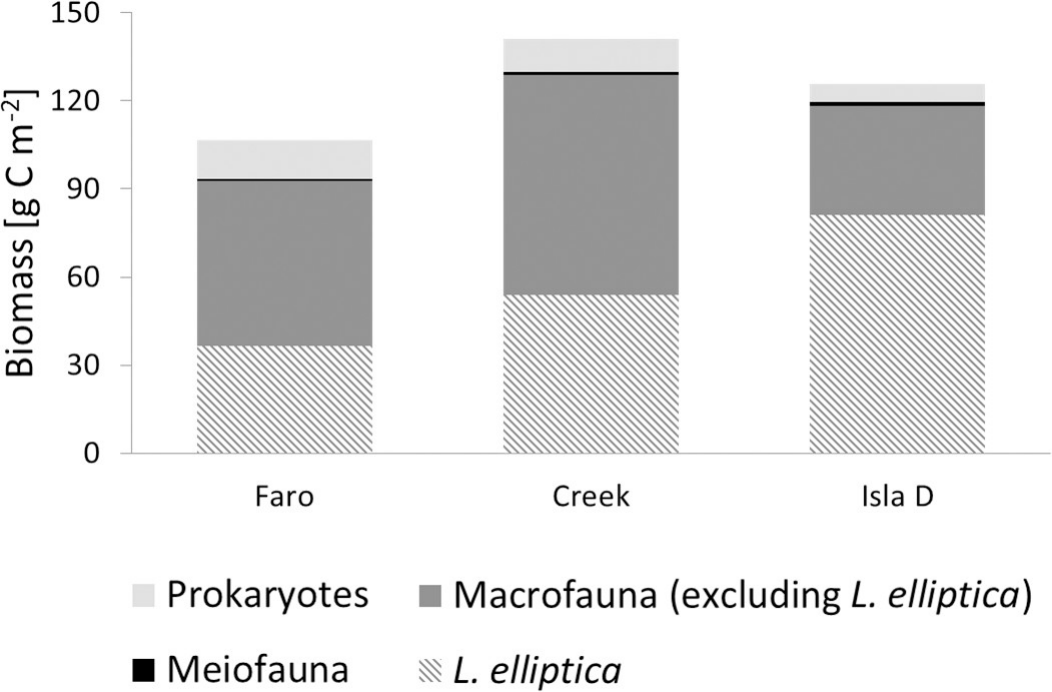
Mean biomasses of prokaryotic, meio- and macrofauna. Macrofauna is the major standing carbon stock in Potter Cove and the bivalve *Laternula elliptica* contributes a large portion to the total macrofauna biomass. In order to compare prokaryotic biomass with the biomasses of other biota size classes, it is expressed as densities per unit surface area.

At Isla D, meiofauna biomass, and macrofauna biomass (the latter excluding *L*. *elliptica*; 1522 ± 240 μg C 10 cm^-2^, 37 ± 33 g C m^-2^, respectively) were similar to the values reported for Faro and Creek (Fig 2B, S5 Table). The macrofauna density (excluding *L*. *elliptica*; 3074 ± 815 ind. m^-2^) at Isla D was similar to Faro, but significantly lower compared to Creek (Fig 2B, S5 Table). Furthermore, prokaryotic density prokaryotic biomass, and *L*. *elliptica* individual biomass (4.2 ± 1.2 × 10^9^ cells cm^-^^3^ sediment, 0.12 ± 0.02 mg C cm^-3^ sediment^-1^, 0.29 ± 0.10 g C ind.^-1^, respectively) were significantly lower compared to Faro and Creek, whereas meiofauna density, *L*. *elliptica* density, and *L*. *elliptica* biomass (3799 ± 719 ind. 10 cm^-2^, 276 ± 50 ind. m^-2^, and 81 ± 15 g C m^-2^, respectively) were significantly higher (Fig 2B, S5 Table). The macrofauna community carbon stock made up >90% of the entire community carbon stock at each location and *L*. *elliptica* contributed 39, 42 and 69% to the total macrofauna biomass at Faro, Creek and Isla D, respectively (Fig 3).

Meiofauna density was dominated by nematodes (98, 87, and 99% at Faro, Creek, and Isla D, respectively). Furthermore, macrofauna density (excluding *L*. *elliptica*) at Faro was dominated by the cumacean family *Leuconidae* sp. (Sars, 1878), at Creek by the bivalve *Mysella* sp. (Angas, 1877), and at Isla D by the burrowing bivalve *Aequiyoldia eightsii* (Jay, 1839), while macrofauna biomass (excluding *L*. *elliptica*) was dominated by *Aequiyoldia eightsii* at each location (87, 81, 74% at Faro, Creek, and Isla D, respectively). The Shannon-Wiener diversity index for meiofauna and macrofauna differed only between Creek and Isla D, whereas the meiofauna taxon and macrofauna species richness did not differ between locations (Fig 2B, S5 Table). The BPc of the macrofauna community did not differ among the three locations (Fig 2B, S5 Table).

### Biogeochemical fluxes at the sediment-water interface

Total fluxes, as determined by *in situ* chamber incubations, showed no differences between transparent and black chambers (S4 Table). Therefore, fluxes from transparent and black chambers at each location were pooled. In general, only benthic oxygen influxes were measured in Potter Cove (from the water column to the seafloor). The TOU at Creek (43 ± 9 mmol O_2_ m^-2^ d^-1^) exceeded Isla D′s TOU (18 ± 3 mmol O_2_ m^-2^ d^-1^) significantly, while at Faro the TOU (33 ± 11 mmol O_2_ m^-2^ d^-1^) did not differ significantly from the TOU of Creek and Isla D (Fig 2C, S5 Table). *In situ* measured oxygen profiles had an oxygen penetration depth of 3–8 mm and a DOU that ranged from 1.5 to 2.4 mmol O_2_ m^-2^ d^-1^ (Fig 2C, S5 Table). The DOU made up 5.0% of the TOU at Faro, 3.5% at Creek and 13.0% at Isla D. The C-TOU was 25 ± 9, 33 ± 7, and 11 ± 6 mmol C m^-2^ d^-1^ at Faro, Creek, and Isla D, respectively.

The total DIC efflux was 12–23 mmol DIC m^-2^ d^-1^, and the diffusive DIC efflux was 0.1–0.5 mmol DIC m^-2^ d^-1^. Both DIC fluxes, total and diffusive, did not differ between the locations (S5 Table). The sediment respiration quotient (RQ = │total DIC flux│/TOU) was 0.55, 0.53, and 0.65 for Faro, Creek, and Isla D, respectively.

Total and diffusive fluxes of phosphate, ammonium, and nitrite were effluxes (from the sediment to the water column), whereas the nitrate flux was an influx (into the sediment). The highest total flux of each nutrient was measured at Creek and the lowest at Isla D, which differed significantly from each other (Fig 2C, S5 Table). Total nutrient fluxes at Faro were either similar to both other locations (total phosphate efflux and total ammonium efflux) or only similar to Isla D and differed significantly from Creek (total nitrite efflux and nitrate influx (Fig 2C, S5 Table)). The diffusive ammonium efflux and the diffusive nitrite net efflux were similar at the three locations. However, the diffusive phosphate efflux was highest at Faro and differed significantly from Creek, while the diffusive nitrate uptake was significantly lower at Creek compared to Faro and Isla D (Fig 2C, S5 Table). The diffusive nutrient fluxes contributed only little to the total nutrient fluxes. Diffusive phosphate fluxes contributed 11, 1 and 13% to the total phosphate flux; diffusive ammonium fluxes contributed 3, 0.6 and 6% to the total ammonium fluxes; diffusive nitrite fluxes contributed 1, 0.3 and 4.3% to the total nitrite fluxes; and diffusive nitrate fluxes contributed 9, 2 and 13% to the total nitrate fluxes at Faro, Creek and Isla D, respectively.

### Predictors of biogeochemical fluxes at the sediment-water interface

**Fig 4:**
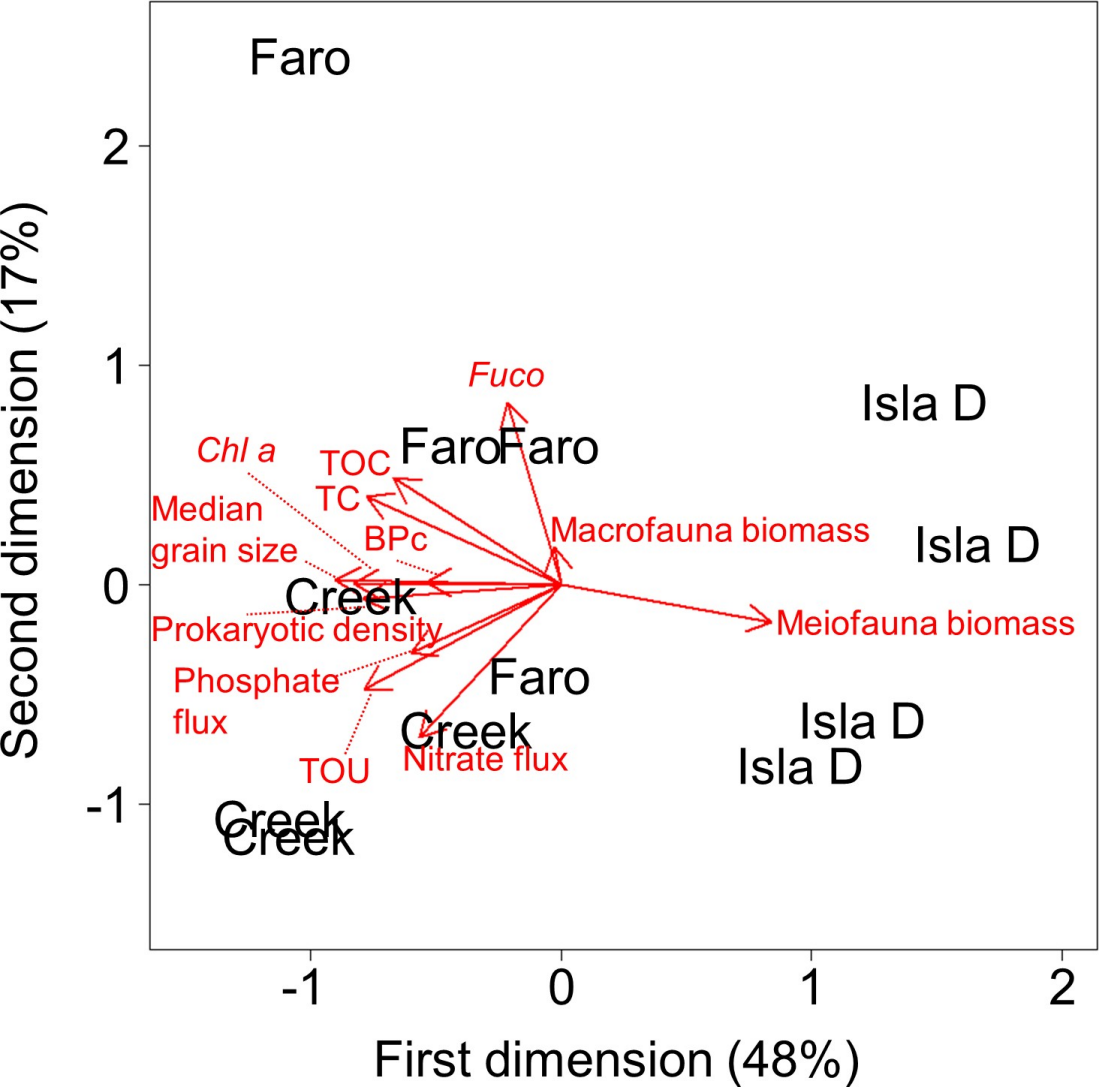
PCA results. Each parameter represents several measured and strongly correlated parameters (r = 0.8, S4 Table). The angles between the arrows of two parameters represent relations ranging between total dependence (0° angle) and total independence (90° angle). Faro, Creek and Isla D display different habitats within Potter Cove, with Faro and Creek showing a small overlap (= similarities). The PCA was conducted in the scaling two-mode on a subset of replicate values (see ‘Materials and methods’).

The results of the PCA revealed that Isla D was a distinct habitat within Potter Cove, while Faro and Creek showed some overlap (Fig 4). The first dimension mainly represented median grain size, meiofauna biomass, and *Chl a*, (Eigenvalues: -899, 0.841, -0.827, respectively), and it distinguished Faro and Creek from Isla D. The second dimension mainly represented *Fuco*, the total nitrate flux, and TOC (Eigenvalues: 0.831, -0.693, 0.486, respectively), and it separated Faro from Creek. The median grain size was positively correlated with *Chl a*, BPc and the prokaryotic density but negatively with meiofauna biomass. TC and TOC, as well as TOU, total phosphate fluxes, and total nitrate fluxes, were well correlated with each other, respectively. Macrofauna biomass was correlated with *Fuco*, however, due to the short length of the macrofauna biomass arrow, its influence on the separation of the locations can be considered low. It needs to be mentioned that each parameter also represents other correlated parameters (S4 Table).

The linear model revealed that the TOU was best predicted by *Chl a*, which explains 74% of the variability in TOU (p < 0.001). The total ammonium and nitrite fluxes were best predicted by *Phaeo* (57% variability explained, p = 0.002) and *Chl a* (77% variability explained, p < 0.001), respectively, while the total nitrate flux was best predicted by the combination of *Fuco* and *Phaeo* (53% variability explained, p = 0.013). The linear model for the total phosphate fluxes indicated *Chl a* as predictive parameter but was not significant (p = 0.057) and explained only 25% of the total phosphate flux variability.

## Discussion

### The influence of glacial melt processes on Antarctic shallow benthic mineralization

Particle release and ice scour are two consequences of glacial melting, besides others, and both can cause increased turbidity and thus increased sedimentation rates [10, 21, 22, 24, 26]. Based on suspended particulate matter [37], turbidity [30] and sediment accumulation [33] in combination with our measured sediment properties (Fig 2A), Faro, Creek, and Isla D are located in areas of low, intermediate, and high influence of glacial melt-related effects, respectively.

Benthic mineralization at the investigated locations seems to be influenced by the ongoing glacial melt effects. All total fluxes (except for DIC) at the highly influenced location Isla D were lower compared to the intermediately influenced location Creek. At Faro, however, TOU, total phosphate fluxes, and total ammonium fluxes did not differ from the other two locations and total nitrite fluxes and total nitrate fluxes differed only from fluxes at Creek. Our linear model approach revealed that the best predictive parameter explaining the TOU pattern is *Chl a*, while the pattern of total nutrient fluxes are explained by *Chl a*, *Fuco*, and *Phaeo*. Therefore, parameters representing organic matter input are drivers of the benthic organic matter mineralization in Potter Cove during our campaign. This existing relationship between organic matter availability and benthic mineralization rates is not surprising and has been described for other regions, e.g. in the Fram Strait, the Atlantic Ocean, and in the shelf regions of the Chukchi and the Bering Sea [6, 7, 69].

Increasing sedimentation rates and the related increase in turbidity can lower the light availability and consequently reduce pelagic primary production [70]. Sediment coverage of microphytobenthos (MPB) can also reduce benthic primary production [71]. Therefore, the highest food availability was expected at Faro where usually clearer waters are present [30, 37]. However, high *Chl a* values were also measured at Creek. The unexpected high food availability at Creek, where light conditions are less ideal for primary producers due to glacial melt-related particle input [30, 33, 37, 70], might be explained by the supply of bio-available iron via run-off from two creeks close to the location [37]. This might stimulate primary production in the water column and at the seafloor and thus boost benthic mineralization.

A strong relationship between the benthic macrofauna standing stock and benthic mineralization rates was observed in coastal, shallow regions worldwide [4, 5, 51, 72, 73]. In the present study, we also expected a large role of benthic macrofauna in benthic mineralization in Potter Cove, owing to the high contribution of macrofauna biomass to the total benthic biomass (Fig 3) and to the large difference between TOU and DOU (the latter is only mediated by microorganisms) indicating fauna respiration and fauna-mediated oxygen uptake (S5 Table), we also expected a large role of benthic macrofauna in benthic mineralization in Potter Cove. However, macrofauna biomass had low explanatory power for benthic mineralization (Fig 4), and significant differences were only found in benthic macrofauna densities between the intermediately-influenced location Creek and the highly-influenced location Isla D (Fig 2B, S5 Table). The latter ruling out macrofauna densities as an explanatory variable for the observed TOU differences between the three sites. Finally, our model approach did not identify any macrofauna parameters as predictive for benthic mineralization. There are several, likely reasons for our model being unable to identify a relationship between macrofauna and mineralisation: (1) the high patchiness of the benthic macrofauna [33], resulting in high variability between replicates and hence masking differences between locations; (2) suppressed individual respiration rates in areas close to the glacier front (observed for suspension-deposit feeders in Potter Cove, including *L*. *elliptica* [28, 29]) where biomass was high; and (3) the smaller sized individuals of *L*. *elliptica* which by burrowing at shallower depths might have limited oxygenation of the deeper sediment and the related deep burial of organic matter, resulting in reduced microbial respiration [74, 75]. Furthermore, the differences in time since the investigated locations became glacial ice-free might have an additional influence since it contributes to small scale functional differences between the three assemblages which are under different successional stages [33].

It has to be considered that our study represents only a small area in Potter Cove. Recently, 10 sub-habitats were identified in Potter Cove, delineated based on 42 benthic environmental parameters [76]. These sub-habitats, in which Faro, Creek and Isla D are located, extend over several hundred square meters, experience different intensities of lithogenic input from the melting Fourcade glacier, and thus confirms that the three investigated locations are representative for areas experiencing different intensities of glacial melt-related effects. Despite its limitations, our study shows that ongoing glacial melt-related effects can locally impact benthic mineralization processes.

### Spatial variability of benthic biogeochemical fluxes at shallow coasts of the Western Antarctic Peninsula

The TOU measured in this study was of the same order of magnitude as TOU values found at Signy Island at 8–9 m depth in austral summer (20–90 mmol O_2_ m^-2^ d^-1^) [13] and Marian Cove at 30 m depth (12–36 mmol O_2_ m^-2^ d^-1^) [14]. However, the oxygen penetration depth was up to four times deeper than the reported 2–3 mm for Signy Island [13]. This difference could also be related to temporal variability, since the inter-annual differences between benthic oxygen fluxes can be large, e.g. 25 mmol O_2_ m^-2^ d^-1^ in February 1991 and 60 mmol O_2_ m^-2^ d^-1^ in February 1992, although the organic matter supply was similar [13]. The studies [13] and [14] investigated the benthic oxygen flux at one location within their study site. Our study provides the first insights into the small-scale spatial variability of benthic oxygen fluxes in shallow coastal Antarctic sediments. Within a radius of less than one kilometer, total and diffusive benthic oxygen fluxes can vary 2–3 -fold, which is similar to seasonal variations [13]. This might be a result of the heterogeneous distribution of different habitats (Fig 4) in Potter Cove [33].

The respiration quotient (RQ) of Potter Cove’s benthic community is, with less than 0.7, unusually low and indicates that much more oxygen is consumed than DIC released. The RQ value might be biased by differences in the precision of DIC and oxygen concentration analyses, which are the basis for the RQ calculation, with Winkler titration for the oxygen concertation measurement as the more precise method. However, such low RQs were also reported in the temperate Boston Harbor region [77] and an Arctic fjord [78]. The low RQs in these studies are explained by high faunal abundances and low values of near-surface sulfide concentrations [77], which were also found in Potter Cove [33, 79]. It is important to note that the low RQs were reported for the winter season [78], while our samples were collected in summer.

Our nutrient fluxes were in a similar range as those measured in the neighboring Marian Cove at 30 m water depth and during springtime, except for 3–4 times higher ammonium fluxes at Creek than those measured in Marian Cove [14]. This indicates that nutrient fluxes measured at different depths within the spring and summer period can be in the same range at King George Island, although ammonium fluxes have shown significant variation at Creek. Furthermore, the sulfate concentration in pore water profiles at the three locations Faro, Creek and Isla D was constant in the first 10 cm (S2 Fig), indicating the absence of sulfide in this sediment depth and a deep aerobic and suboxic sediment layer. This is similar to findings of other investigations in Potter Cove [79].

### Supply of the benthic carbon demand in Potter Cove

The benthic carbon demand combined with primary production data can be used to assess whether a habitat or ecosystem is in an auto- or heterotrophic state. It was suggested that the water column production in Potter Cove would probably not be sufficient to nourish the benthic community [80]. The total pelagic primary production between October 1991 and February 1992 ranged between 236–259 mg C m^-2^ d^-1^ (= 19.7–21.6 mmol C m^-2^ d^-1^) [80] and was on average constant over the period 1991–2009 [36]. Since 2009, however, the monthly pelagic *Chl a* concentration increased 2 to 3-fold, compared to the mean *Chl a* concentration from 1992 to 2016 [70]. This would be sufficient to nourish the benthic carbon demand at Faro, Creek, and Isla D. However, the findings on pelagic primary production [80] and on pelagic *Chl a* concentrations [70] are based on measurements at two and three stations, respectively, located in the inner and outer Potter Cove. In contrast, our study resolves spatial variability at three locations only in the inner part of Potter Cove. In any case, the pelagic primary production appeared to be able to feed the benthic carbon demand during the sampling period of this study. This indicates Potter Cove might be an autotrophic ecosystem during the summer months.

Other carbon sources such as microorganisms, macroalgae debris, and the microphytobenthos are also likely to supply the benthic carbon demand in Potter Cove [80]. Brownish MPB mats were observed by SCUBA divers at Faro, Creek and Isla D within Potter Cove (S2 Fig), which are known to form intense blooms in the neighboring Marian Cove [81]. The relatively high values of *Fuco* (S5 Table) indicate that diatoms constituted most of the MPB community in Potter Cove, similar to the findings of Al-Handal and Wulff [82]. There was no difference in the TOU between transparent and black chambers (S4 Table). Thus, the MPB assemblage enclosed in the used benthic chambers was unable to cover the entire benthic carbon demand. This might be a result of the high turbidity which usually develops during austral summer [30] and likely limited MPB primary production. However, the findings of Hoffmann et al. [unpublished] indicate that MPB at Faro and Creek has the potential to supply substantial organic carbon for the benthic carbon demand.

In coastal areas, the spatial variability of the benthic carbon demand is closely related to the benthic carbon supply by primary producers ([51], this study). Primary production, however, is influenced by light [80] and thus it may be influenced by glacial melt-related effects. With ongoing loss of Antarctic shelf ice [17] and ongoing retreat of glaciers [19], vast shallow coastal areas will eventually face alterations in benthic mineralization owing to (1) the influence of an increased particle release and related effects [this study]; (2) succession of newly ice-free areas by benthic assemblages [30–33];(3) changes in the benthic community structure [28–32]; and (4) metabolic adaptive responses of the benthic community to sedimentation [28, 29]. Nevertheless, differences in benthic mineralization will ultimately depend on the pace of climatic changes [83] and the related changes in organic matter input by primary production and the intensity of glacial melt-related processes on a local scale.

## Supporting information

S1 Fig Nutrient concentration profiles from pore water extractions. (TIFF)

S2 Fig Photos of brownish microphytobenthic (MPB) mats at Faro, Creek and Isla D. The photos demonstrate the occurrence of MPB in Potter Cove.
(TIF)

S1 Table Sediment reworking (Mi) and mobility (Ri) scores for the macrofauna community in Potter Cove in order to calculate the bioturbation potential [46]. Scores were assigned for the lowest possible taxonomic level (Order, Family or Genus). Mi score scale: 1 for organisms that live in fixed tubes, 2 indicates limited movement, 3 indicates slow, free movement through the sediment matrix, and 4 indicates free movement, that is, via a burrow system. Ri score scale: 1 for epifauna, 2 for surficial modifiers, 3 for upward and downward conveyors, 4 for biodiffusors, and 5 for regenerators.
(PDF)

S2 Table Sediment depth across the diffusive nutrient flux was calculated. The basis for the diffusive flux calculation was the change in the nutrient concentration over depth. All calculations started at the -0.5 cm depth, which is the bottom water concentration, and were calculated across the sediment depth given in the table, despite the nitrite influx, which started at the sediment depth at which the nitrite efflux ended.
(PDF)

S3 Table P-values of the Levene's test and Student's t-test, comparing TOUs from black and transparent chamber incubations. (PDF)

S4 Table Result of Pearson correlation. (PDF)

S5 Table Measured mean values ± SD of sediment, biogenic, benthic community and flux parameters. N is given in brackets. The letters a, b, c indicate significant differences (p<0.05) of a parameter between the locations, while NS indicates no significant differences. Furthermore, the p-values of the Shapiro─Wilk test, the Levene's test, ANOVA and Kruskal-Wallis investigations and the associated post-hoc test are given.
(PDF)
