# Supplementary figures and images for: Spatial variability of biogeochemistry in shallow coastal benthic communities of Potter Cove (Antarctica) and the impact of a melting glacier
Source: PLoS One. 2018 Dec 19;13(12):e0207917. doi: 10.1371/journal.pone.0207917 (PMC6300201; doi:10.1371/journal.pone.0207917)

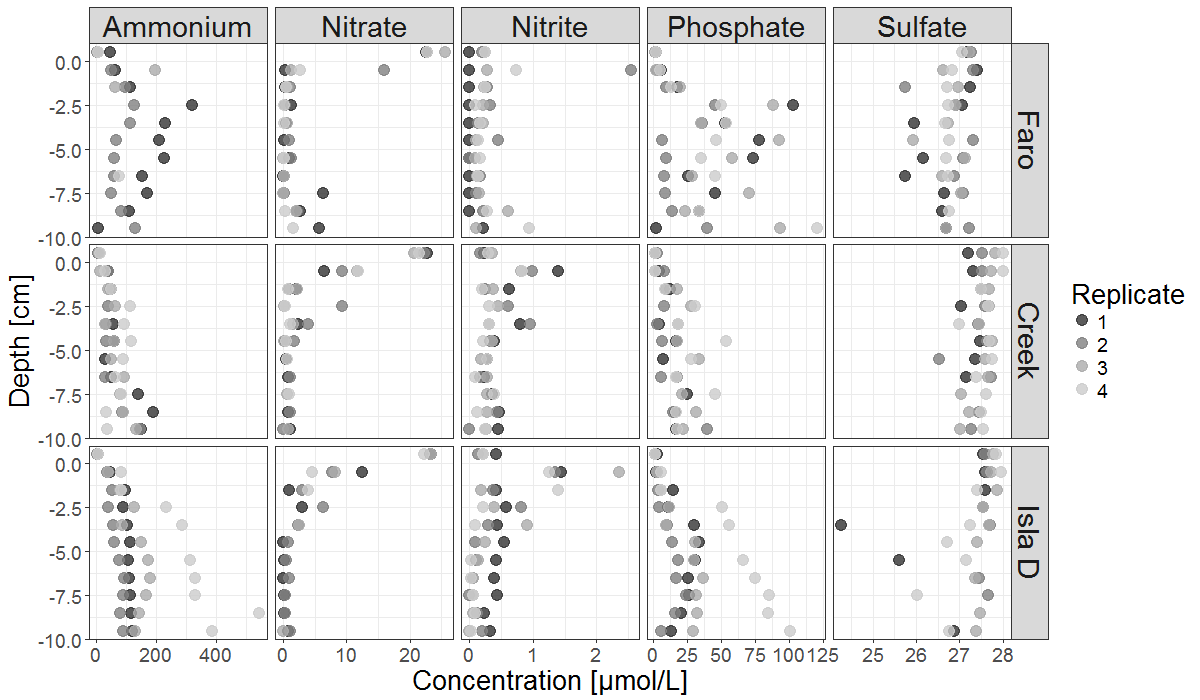

Supplement: S1 Fig — (TIFF) [file pone.0207917.s001.tiff]

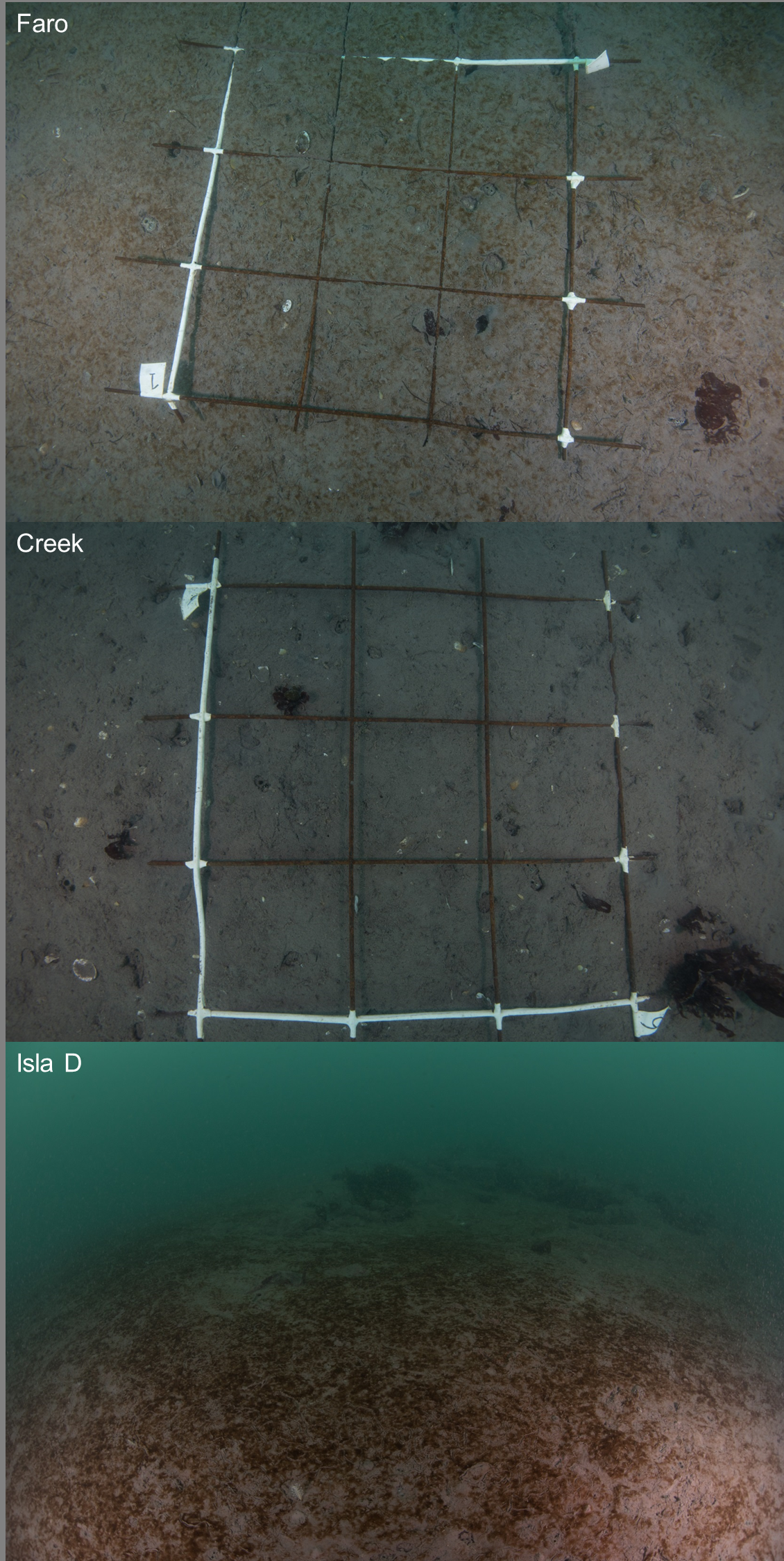

Supplement: S2 Fig — The photos demonstrate the occurrence of MPB in Potter Cove. (TIF) [file pone.0207917.s002.tif]
