# Supplementary material for: Spatial variability of biogeochemistry in shallow coastal benthic communities of Potter Cove (Antarctica) and the impact of a melting glacier
Source: PLoS One. 2018 Dec 19;13(12):e0207917. doi: 10.1371/journal.pone.0207917 (PMC6300201; doi:10.1371/journal.pone.0207917)
Supplement: S1 Table — Scores were assigned for the lowest possible taxonomic level (Order, Family or Genus). Mi score scale: 1 for organisms that live in fixed tubes, 2 indicates limited movement, 3 indicates slow, free movement through the sediment matrix, and 4 indicates free movement, that is, via a burrow system. Ri score scale: 1 for epifauna, 2 for surficial modifiers, 3 for upward and downward conveyors, 4 for biodiffusors, and 5 for regenerators. (PDF) [file pone.0207917.s003.pdf]

**Class**	**Order**	**Family**	**R_i**	**M_i**
Bivalvia	Nuculanoida	Yoldiidae	2	3
	Nuculida	Nuculidae (*Nucula* sp.)	2	3
	Veneroida	Montacutidae (*Mysella* sp.)	2	2
	Lucinoida	Thyasiridae	3	2
	Myoida	Myidae (*Mya* sp.)	2	2
Gastropoda	Littorinimorpha	Rissoidae	2	3
Polychaeta	Phyllodocida	Polynoidae	4	3
		Nephtyidae	4	3
	Terebellida	Cirratulidae	2	2
		Maldanidae	3	1
		Opheliidae (*Travisia* sp., *Ophelina* sp.)	4	3
Malacostra	Amphipoda	Lysianassidae	2	3
		Phoxocephalidae	2	3
		Oedicerotidae	2	3
		Eusiridae	2	3
	Cumacea	Leuconidae (*Eudorella* sp.)	2	3
		Bodotriidae	2	3
		Nannastacidae	2	3
	Tanaidacea	Paratanoidea (incertae sedis)	2	2
	Isopoda		2	3
Ostracoda	Myodocopida		2	3
Priapulida		Priapulidae	4	2
Anthozoa	Pennatulacea		2	2
