# Supplementary material for: Spatial variability of biogeochemistry in shallow coastal benthic communities of Potter Cove (Antarctica) and the impact of a melting glacier
Source: PLoS One. 2018 Dec 19;13(12):e0207917. doi: 10.1371/journal.pone.0207917 (PMC6300201; doi:10.1371/journal.pone.0207917)
Supplement: S2 Table — The basis for the diffusive flux calculation was the change in the nutrient concentration over depth. All calculations started at the -0.5 cm depth, which is the bottom water concentration, and were calculated across the sediment depth given in the table, despite the nitrite influx, which started at the sediment depth at which the nitrite efflux ended. (PDF) [file pone.0207917.s004.pdf]

Station	Replicate	Sediment depth [cm] across diffusive fluxes were calculated				
		Phosphate efflux	Ammonium efflux	Nitrite efflux	Nitrite influx	Nitrate influx
Faro	1	2.5	2.5	0.5	1.5	0.5
	2	2.5	2.5	0.5	1.5	1.5
	3	2.5	1.5	0.5	1.5	0.5
	4	2.5	3.5	0.5	1.5	0.5
Creek	1	2.5	1.5	0.5	1.5	0.5
	2	1.5	1.5	0.5	1.5	0.5
	3	2.5	2.5	0.5	1.5	1.5
	4	4.5	2.5	0.5	1.5	1.5
Isla D	1	4.5	3.5	0.5	1.5	1.5
	2	7.5	1.5	0.5	1.5	0.5
	3	6.5	2.5	0.5	1.5	0.5
	4	3.5	3.5	0.5	2.5	0.5
