# Supplementary material for: Spatial variability of biogeochemistry in shallow coastal benthic communities of Potter Cove (Antarctica) and the impact of a melting glacier
Source: PLoS One. 2018 Dec 19;13(12):e0207917. doi: 10.1371/journal.pone.0207917 (PMC6300201; doi:10.1371/journal.pone.0207917)
Supplement: S3 Table — (PDF) [file pone.0207917.s005.pdf]

	Location	Levene's test	Student' s t-test
**TOU**	Faro	0.73	0.79
	Creek	0.65	0.92
	Isla D	0.36	0.21
**Total DIC efflux**	Faro	not enough observations	
	Creek	<0.05	not enough observations
	Isla D	<0.05	not enough observations
**Total phosphate efflux**	Faro	1.00	0.87
	Creek	0.60	0.78
	Isla D	<0.05	0.46
**Total ammonium efflux**	Faro	0.66	0.67
	Creek	0.36	0.95
	Isla D	0.61	0.74
**Total nitrite efflux**	Faro	0.22	0.83
	Creek	0.91	0.48
	Isla D	<0.05	0.48
**Total nitrate uptake**	Faro	0.48	0.79
	Creek	0.78	0.57
	Isla D	0.30	0.40
