# Supplementary material for: Spatial variability of biogeochemistry in shallow coastal benthic communities of Potter Cove (Antarctica) and the impact of a melting glacier
Source: PLoS One. 2018 Dec 19;13(12):e0207917. doi: 10.1371/journal.pone.0207917 (PMC6300201; doi:10.1371/journal.pone.0207917)
Supplement: S4 Table — (PDF) [file pone.0207917.s006.pdf]

	**Median grain size**	**Porosity**	**Silt fraction <63µm**	***Chl a***	***Fuco***	***Phaeo***	**TC**	**TOC**	**TN**	**TIC**	**Bacteria density**	**Meiofauna density**	**Meiofauna biomass**	**Macrofauna density**	**Macrofauna biomass**	**BPc**	**TOU**	**Total phosphate flux**	**Total ammonium flux**	**Total nitrite flux**	**Total nitrate flux**
**Median grain size**	1																				
**Porosity**	-0.857	1																			
**Silt fraction <63µm**	-0.954	0.946	1																		
Chl a	0.704	-0.678	-0.776	1																	
Fuco	0.18	-0.271	-0.244	0.3	1																
Phaeo	0.693	-0.652	-0.762	0.986	0.198	1															
**TC**	0.853	-0.656	-0.744	0.475	0.401	0.422	1														
**TOC**	0.483	-0.293	-0.404	0.566	0.486	0.487	0.717	1													
**TN**	0.461	-0.283	-0.381	0.527	0.541	0.439	0.742	0.954	1												
**TIC**	0.879	-0.693	-0.772	0.402	0.322	0.363	0.977	0.555	0.594	1											
**Bacteria density**	0.833	-0.531	-0.701	0.505	0.049	0.476	0.742	0.479	0.409	0.758	1										
**Meiofauna density**	-0.787	0.825	0.896	-0.824	-0.344	-0.783	-0.602	-0.489	-0.438	-0.579	-0.613	1									
**Meiofauna biomass**	-0.761	0.778	0.821	-0.659	-0.384	-0.583	-0.737	-0.636	-0.587	-0.693	-0.649	0.926	1								
**Macrofauna density**	0.572	-0.458	-0.614	0.51	-0.079	0.568	0.38	0.259	0.142	0.388	0.44	-0.626	-0.494	1							
**Macrofauna biomass**	-0.122	0.149	0.075	0.363	0.02	0.402	-0.134	0.262	0.267	-0.241	-0.235	0	0.154	0.367	1						
**BPc**	0.489	-0.347	-0.519	0.566	-0.007	0.621	0.339	0.338	0.245	0.315	0.388	-0.535	-0.378	0.939	0.613	1					
**TOU**	0.707	-0.607	-0.73	0.761	-0.14	0.766	0.391	0.271	0.255	0.396	0.606	-0.736	-0.609	0.394	-0.078	0.295	1				
**Total phosphate flux**	-0.527	0.469	0.532	-0.562	0.025	-0.583	-0.358	-0.335	-0.297	-0.327	-0.26	0.46	0.406	-0.193	0.143	-0.065	-0.733	1			
**Total ammonium flux**	-0.676	0.591	0.678	-0.656	-0.082	-0.656	-0.507	-0.326	-0.391	-0.507	-0.406	0.584	0.504	-0.193	0.144	-0.097	-0.848	0.896	1		
**Total nitrite flux**	-0.676	0.597	0.72	-0.832	-0.002	-0.843	-0.367	-0.356	-0.319	-0.339	-0.463	0.72	0.553	-0.485	-0.12	-0.406	-0.916	0.817	0.855	1	
**Total nitrate flux**	0.402	-0.264	-0.408	0.491	-0.46	0.531	0.068	0.142	-0.028	0.058	0.528	-0.53	-0.428	0.534	-0.018	0.41	0.745	-0.444	-0.364	-0.652	1
