# Supplementary material for: Spatial variability of biogeochemistry in shallow coastal benthic communities of Potter Cove (Antarctica) and the impact of a melting glacier
Source: PLoS One. 2018 Dec 19;13(12):e0207917. doi: 10.1371/journal.pone.0207917 (PMC6300201; doi:10.1371/journal.pone.0207917)
Supplement: S5 Table — N is given in brackets. The letters a, b, c indicate significant differences (p<0.05) of a parameter between the locations, while NS indicates no significant differences. Furthermore, the p-values of the Shapiro─Wilk test, the Levene's test, ANOVA and Kruskal-Wallis investigations and the associated post-hoc test are given. (PDF) [file pone.0207917.s007.pdf]

	Faro	Creek	Isla D	Sqrt-Transforma-tion	P-value of Shapiro–Wilk test [Faro, Creek, Isla D]	P-value of Levene's test	Test for analysis of variance and associated p-value	Post-hoc Test	P-value of pairwise post-hoc test [Faro-Creek, Faro-Isla D, Creek-Isla D]
**Mean median grain size [µm]**	116 ± 27 [a] (21)	120 ± 9 [a] (21)	20 ± 30 [b] (13)	-	0.084, 0.481, 0.529	<0.001	ANOVA, <0.001	Games-Howell	0.775, <0.001, <0.001
**Mean portion of silt (<63µm) [%]**	39 ± 5 [a] (21)	28 ± 4 [b] (21)	83 ± 12 [c] (13)	-	0.022, 0.261 0.632	-	Kruskal-Wallis, <0.001	Bonferroni	0.001, 0.006, <0.001
**Mean portion of sand (>63µm) [%]**	61 ± 5 [a] (21)	72 ± 4 [b] (21)	17 ± 12 [c] (13)	-	0.022, 0.261 0.632	-	Kruskal-Wallis, <0.001	Bonferroni	0.001, 0.006, <0.001
**Mean porosity**	0.56 ± 0.08 [a] (21)	0.51 ± 0.05 [a] (21)	0.76 ± 0.12 [b] (14)	-	0.447, 0.167, 0.609	0.040	ANOVA, <0.001	Games-Howell	0.082, <0.001, <0.001
**Mean TC [µg C mg sediment^{-1}]**	7.3 ± 1.4 [a] (56)	2.6 ± 0.5 [b] (38)	5.5 ± 0.9 [c] (46)	-	<0.001, 0.135, 0.074	-	Kruskal-Wallis, <0.001	Bonferroni	<0.001, <0.001, <0.001
**Mean TIC [µg C mg sediment^{-1}]**	4.8 ± 0.8 [a] (54)	0.6 ± 0.3 [b] (38)	3.3 ± 0.4 [c] (46)	-	<0.001, 02.85 <0.001	-	Kruskal-Wallis, <0.001	Bonferroni	<0.001, <0.001, <0.001
**Mean TOC [µg C_{org} mg sediment^{-1}]**	2.3 ± 0.9 [NS] (54)	2.0 ± 0.4 [NS] (38)	2.2 ± 0.8 [NS] (46)	-	<0.001, <0.001, 0.001,	-	Kruskal-Wallis, 0.260	-	-
**Mean TN [µg N mg sediment^{-1}]**	0.51 ± 0.24 [a] (56)	0.37 ± 0.08 [b] (38)	0.45 ± 0.16 [ab] (46)	-	<0.001, 0.205, 0.007,	-	Kruskal-Wallis, 0.007	Bonferroni	0.005, 0.789, 0.139
**Mean TOC/TC [%]**	32 ± 8 [a] (54)	78 ± 11 [b] (38)	39 ± 10 [c] (46)	-	0.051, 0.321, 0.016	-	Kruskal-Wallis, <0.001	Bonferroni	<0.001, 0.025, <0.001

**Mean *Chl a* [µg g sediment^{-1}]**	6.3 ± 4.6 ^{a} (28)	11.3 ± 9.3 ^{a} (22)	3.0 ± 1.4 ^{b} (21)	X	0.123, 0.057, 0.735	<0.001	ANOVA, <0.001	Games-Howell	0.105 0.003 0.001
**Mean *Fuco* [µg g sediment^{-1}]**	3.1 ± 2.6 ^{a} (28)	6.6 ± 5.7 ^{b} (22)	1.3 ± 0.9 ^{a} (21)	X	0.100, 0.071, 0.168	<0.001	ANOVA, <0.001	Games-Howell	0.038, <0.001, 0.007
**Mean *Phaeo* [µg g sediment^{-1}]**	2.4 ± 0.8 ^{NS} (28)	1.8 ± 1.0 ^{NS} (22)	1.9 ± 0.7 ^{NS} (21)	-	0.282, 0.272, 0.006	-	Kruskal-Wallis, 0.037	Bonferroni	0.150, 0.056, 1.000
**Mean *Chl*/*Phaeo***	2.3 ± 1.2 ^{a} (28)	6.9± 5.3 ^{b} (22)	1.6 ± 0.6 ^{a} (21)	-	0.038, 0.007, 0.220	-	Kruskal-Wallis, <0.001	Bonferroni	<0.001, 0.19, <0.001
**Mean prokaryotic density [10^{9} cells cm^{-3} sediment^{-1}]**	6.1 ± 1.2 ^{a} (36)	6.0 ± 2.1 ^{a} (46)	4.2 ± 1.2 ^{b} (30)	X	0.521, 0.456, 0.293	0.057	ANOVA, <0.001	Tukey	0.803, <0.001, <0.001
**Mean prokaryotic biomass [mg C cm^{-3} sediment^{-1}]**	0.26 ± 0.02 ^{a} (8)	0.22 ± 0.02 ^{b} (10)	0.12 ± 0.02 ^{c} (8)	-	0.656, 0.232, 0.529	0.456	ANOVA, <0.001	Tukey	0.001, <0.001, <0.001
**Mean meiofauna density [Ind. 10 cm^{-2}]**	2368 ± 471 ^{a} (5)	1524 ± 231 ^{a} (5)	3799 ± 719 ^{b} (5)	-	0.195, 0.862, 0.575	0.351	ANOVA, <0.001	Tukey	0.090, 0.005, <0.001
**Mean meiofauna biomass [µg C 10 cm^{-2}]**	990 ± 190 ^{ab} (5)	980 ± 204 ^{a} (5)	1522 ± 240 ^{b} (5)	-	0.165, 0.992, 0.848	0.931	ANOVA, 0.005	Tukey	0.997, 0.105, 0.009
**Mean macrofauna density [Ind. m^{-2}], without *L. elliptica***	33574 ± 24902 ^{ab} (4)	65612 ± 35948 ^{a} (4)	3074 ± 815 ^{b} (4)	-	0.446, 0.616, 0.760	0.073	ANOVA, 0.042	Tukey	0.313, 0.344, 0.034
**Mean macrofauna biomass [g C m^{-2}], without *L. elliptica***	56 ± 39 ^{NS} (4)	75 ± 26 ^{NS} (4)	37 ± 32 ^{NS} (4)	-	0.456, 0.368, 0.123	0.830	ANOVA, 0.403	-	-
**Mean *L. elliptica* density [Ind. m^{-2}]**	93 ± 26 ^{a} (15)	157 ± 44 ^{b} (16)	276 ± 50 ^{c} (16)	-	0.211, 0.335, 0.662	0.071	ANOVA, <0.001	Tukey	<0.001, <0.001, <0.001

**Mean *L. elliptica* biomass [g C m^{-2}]**	36 ± 9 ^{a} (15)	54 ± 16 ^{b} (16)	81 ± 15 ^{c} (16)	X	0.208, 0.477, 0.059	0.637	ANOVA, <0.001	Tukey	0.001, <0.001, <0.001
**Mean individual *L. elliptica* biomass [g C ind.$^{-1}$]**	0.39 ± 0.16 ^{a} (281)	0.34 ± 0.14 ^{b} (458)	0.29 ± 0.10 ^{c} (887)	-	0.236, <0.001, <0.001	-	Kruskal-Wallis, <0.001	Bonferroni	<0.001, <0.001, <0.001
**S Meiofauna**	7.8 ± 1.7 ^{NS} (5)	7.0 ± 1.4 ^{NS} (5)	6.6 ± 0.5 ^{NS} (5)	-	0.928, 0.967, NS	-	Kruskal-Wallis, 0.440	-	-
**H' Meiofauna**	0.12 ± 0.03 ^{ab} (5)	0.44 ± 0.10 ^{a} (5)	0.08 ± 0.01 ^{b} (5)	-	0.332, 0.830, 0.011	-	Kruskal-Wallis, 0.002	Bonferroni	0.198, 0.312, 0.002
**S Macrofauna**	13.3 ± 0.8 ^{NS} (4)	14 ± 1.2 ^{NS} (4)	13.5 ± 1.1 ^{NS} (4)	-	0.273, 0.161, 0.972	0.856	ANOVA, 0.693	-	-
**H' Macrofauna**	1.3 ± 0.2 ^{ab} (4)	1.2 ± 0.2 ^{a} (4)	1.7 ± 0.1 ^{b} (4)	-	0.5471, 0.129, 0.039	-	Kruskal-Wallis, 0.023	Bonferroni	1.000, 0.093, 0.032
**BPc**	168540 ± 97270 ^{NS} (4)	270960 ± 105813 ^{NS} (4)	99444 ± 37279 ^{NS} (4)	-	0.757, 0.198, 0.672	0.419	ANOVA, 0.098	-	-
**BPc *L. elliptica***	10369 ± 2727 ^{a} (15)	16379 ± 4537 ^{b} (16)	26767 ± 4839 ^{c} (16)	-	0.199, 0.967, 0.114	0.229	ANOVA, <0.001	Tukey	<0.001, <0.001, <0.001
**Oxygen penetration depth [mm]**	4.5-6 (3)	8 (1)	3.5, 4 (2)	-	-	-	-	-	-
**TOU [mmol O_2 m^{-2}d^{-1}]**	33 ± 11 ^{ab} (6)	43 ± 9 ^{a} (6)	18 ± 3 ^{b} (5)	-	0.262, 0.783, 0.094	0.123	ANOVA, 0.003	Tukey	0.187, 0.065, 0.002
**Total DIC flux [mmol DIC m^{-2}d^{-1}]**	-18 ± 3 ^{NS} (2)	-23 ± 8 ^{NS} (6)	-12 ± 5 ^{NS} (5)	-	NS, 0.237, 0.698	-	Kruskal-Wallis, 0.084	-	-

**Total phosphate flux<br>[µmol PO_4 m^{-2}d^{-1}]**	-119 ± 106 ^{ab}<br>(6)	-345 ± 187 ^{a}<br>(6)	-64 ± 46 ^{b}<br>(5)	-	0.073,<br>0.855,<br>0.003	-	Kruskal-<br>Wallis,<br>0.029	Bonferroni	0.119,<br>1.000,<br>0.041
**Total ammonium flux<br>[mmol NH_4 m^{-2}d^{-1}]**	-4.2 ± 2.2 ^{ab}<br>(6)	-7.8 ± 2.8 ^{a}<br>(6)	-2.5 ± 0.7 ^{b}<br>(5)	X	0.061,<br>0.523,<br>0.628	0.695	ANOVA,<br>0.005	Tukey	0.051,<br>0.370,<br>0.004
**Total nitrite flux<br>[µmol NO_2 m^{-2}d^{-1}]**	-62 ± 18 ^{a}<br>(6)	-140 ± 33 ^{b}<br>(6)	-36 ± 12 ^{a}<br>(5)	-	0.865,<br>0.354,<br>0.819	0.200	ANOVA,<br><0.001	Tukey	<0.001,<br>0.259,<br><0.001
**Total nitrate flux<br>[mmol NO_3 m^{-2}d^{-1}]**	0.6 ± 0.1 ^{a}<br>(6)	1.2 ± 0.2 ^{b}<br>(6)	0.5 ± 0.3 ^{a}<br>(5)	-	0.344,<br>0.893,<br>0.443	0.364	ANOVA,<br><0.001	Tukey	0.003,<br>0.811,<br>0.001
**DOU [mmol O_2 m^{-2}d^{-1}]**	1.6 ± 0.4 ^{NS}<br>(3)	1.5 ^{NS}<br>(1)	2.4 ± 0.3 ^{NS}<br>(2)	-	0.943,<br>NS,<br>NS	-	Kruskal-<br>Wallis,<br>0.240	-	-
**Diffusive DIC flux<br>[mmol DIC m^{-2}d^{-1}]**	-0.3 ± 0.1 ^{NS}<br>(4)	-0.1 ± 0.04 ^{NS}<br>(2)	-0.5 ± 0.2 ^{NS}<br>(4)	-	0.515,<br>NS,<br>0.974	-	Kruskal-<br>Wallis,<br>0.057	-	-
**Diffusive phosphate flux<br>[µmol PO_4 m^{-2}d^{-1}]**	-13.5 ± 4.7 ^{a}<br>(4)	-3.5 ± 1.2 ^{b}<br>(4)	-8.0 ± 5.0 ^{ab}<br>(4)	-	0.225,<br>0.415,<br>0.321	0.213	ANOVA,<br>0.039	Tukey	0.031,<br>0.387,<br>0.259
**Diffusive ammonium flux<br>[mmol NH_4 m^{-2}d^{-1}]**	-120 ± 52 ^{NS}<br>(3)	-43 ± 9 ^{NS}<br>(4)	-155 ± 72 ^{NS}<br>(4)	-	0.311,<br>0.428,<br>0.289	0.285	ANOVA,<br>0.079	-	
**Diffusive nitrite net flux<br>[µmol NO_2 m^{-2}d^{-1}]**	-0.8 ± 0.5 ^{NS}<br>(2)	-0.4 ± 0.4 ^{NS}<br>(4)	-1.6 ± 0.7 ^{NS}<br>(4)	-	NS,<br>0.419,<br>0.933	-	Kruskal-<br>Wallis,<br>0.110	-	-
**Diffusive nitrate flux<br>[µmol NO_3 m^{-2}d^{-1}]**	55 ± 17 ^{a}<br>(4)	25 ± 7 ^{b}<br>(4)	68 ± 14 ^{a}<br>(4)	-	0.238,<br>0.576,<br>0.386	0.716	ANOVA,<br>0.009	Tukey	0.049,<br>0.008,<br>0.496
